# Clonal evolution in healthy and premalignant tissues: implications for early cancer interception strategies

**DOI:** 10.1158/1940-6207.CAPR-22-0469

**Published:** 2023-03-17

**Authors:** Jayant K. Rane, Alexander M. Frankell, Clare E. Weeden, Charles Swanton

**Affiliations:** 1 University College London Cancer Institute, London, UK; 2 Department of Clinical Oncology, University College London Hospitals, London, UK; 3 Cancer Evolution and Genome Instability Laboratory, The Francis Crick Institute, London, UK; 4 Cancer Research UK Lung Cancer Centre of Excellence, University College London Cancer Institute, London, UK; 5 Department of Medical Oncology, University College London Hospitals, London, UK

## Abstract

Histologically normal human tissues accumulate significant mutational burden with age. The extent and spectra of mutagenesis are comparable both in rapidly proliferating and post-mitotic tissues and in stem cells compared to their differentiated progeny. Some of these mutations provide increased fitness, giving rise to clones which, at times, can replace the entire surface area of tissues. Compared to cancer, somatic mutations in histologically normal tissues are primarily single nucleotide variations. Interestingly though, the presence of these mutations and positive clonal selection in isolation remains a poor indicator of potential future cancer transformation in solid tissues. Common clonally expanded mutations in histologically normal tissues also do not always represent the most frequent early mutations in cancers of corresponding tissues, indicating differences in selection pressures. Preliminary evidence implies that stroma and immune system co-evolve with age, which may impact selection dynamics. In this review, we will explore the mutational landscape of histologically normal and pre-malignant human somatic tissues in detail and discuss cell-intrinsic and environmental factors that can determine the fate of positively selected mutations within them. Precisely pinpointing these determinants of cancer transformation would aid development of early cancer interventional and prevention strategies.

## Introduction

In 1914, Theodor Boveri hypothesised that irreparable defect in chromosomes leads to the malignant transformation (1). Experimental evidence in 1970s-80s suggested that the acquisition of stepwise mutational events in a normal cell can cause transformation into an indefinitely dividing cell (2–4). Subsequent genomic studies found that all tumour cells share a set of genomic mutations, implying a single, most recent common ancestor (MRCA) (5). The genome of MRCA progeny continues to evolve as it expands in response to rapidly changing cell-intrinsic and cell-extrinsic factors and as a result of neutral genetic drift, resulting in considerable intra-tumour heterogeneity (ITH) (6). ITH is associated with tumour aggressiveness, response to treatment and ultimately, patient outcome (7–14). Such somatic mutational diversity and clonal heterogeneity is now considered as one of the fundamental hallmarks of cancer (15). However in addition, clonally expanded cancer-associated gene mutations have now been identified in multiple histologically normal human tissues (16). Investigating the fate of mutant clones and their interactions with surrounding tissue could inform key issues in cancer medicine including; whether positively selected mutations in histologically normal tissues preferentially transform into cancer, what the barriers are for malignant transformation of ‘initiated’ cells, and whether environmental exposures and germline variations co-operate with somatic mutagenesis during cancer initiation. We will examine pertinent literature regarding the mutational landscape of clones within histologically normal and pre-malignant human tissues and discuss how these insights may in the future be applied to clinical decision making around early cancer detection and prevention.

## Somatic mutations and clonality in histologically normal tissue

Genomic sequencing-based analyses of clonal evolution in cancer and pre-invasive disease have suggested the possibility of clonal expansions even prior to malignant transformation (17,18). The possibility of positive mutational selection leading to clonal expansion in histologically normal tissue was supported by observations of regions of clonal expansions carrying skewed X-inactivation, specific mitochondrial enzyme inactivation, and shared *TP53* inactivation patterns (19). The common challenges associated with detailed clonal analysis within histologically normal tissues such as extremely small clonal size, low DNA input during library preparation, and presence of low frequency alterations have been overcome in the last decade using inventive sample collection and sequencing strategies (20–37). These strategies include the use of micro biopsies, sequencing of single clonal units (e.g., crypts), *in-vitro* expansion of single cells (e.g., organoids), single cell DNA sequencing, and NanoSeq (20–22,26,33). DNA-sequencing of histologically normal tissue using these strategies is providing unequivocal evidence for the presence of widespread somatic mutations and the positive selection of some of these mutations leading to clonal expansion employing methods such as dNdScv (38). This method employs trinucleotide context-dependent substitution matrices to identify positively selected mutations within genes over and above the background mutational changes in the genome.

## Origins of somatic mutations in histologically normal tissue

**Figure 1:**
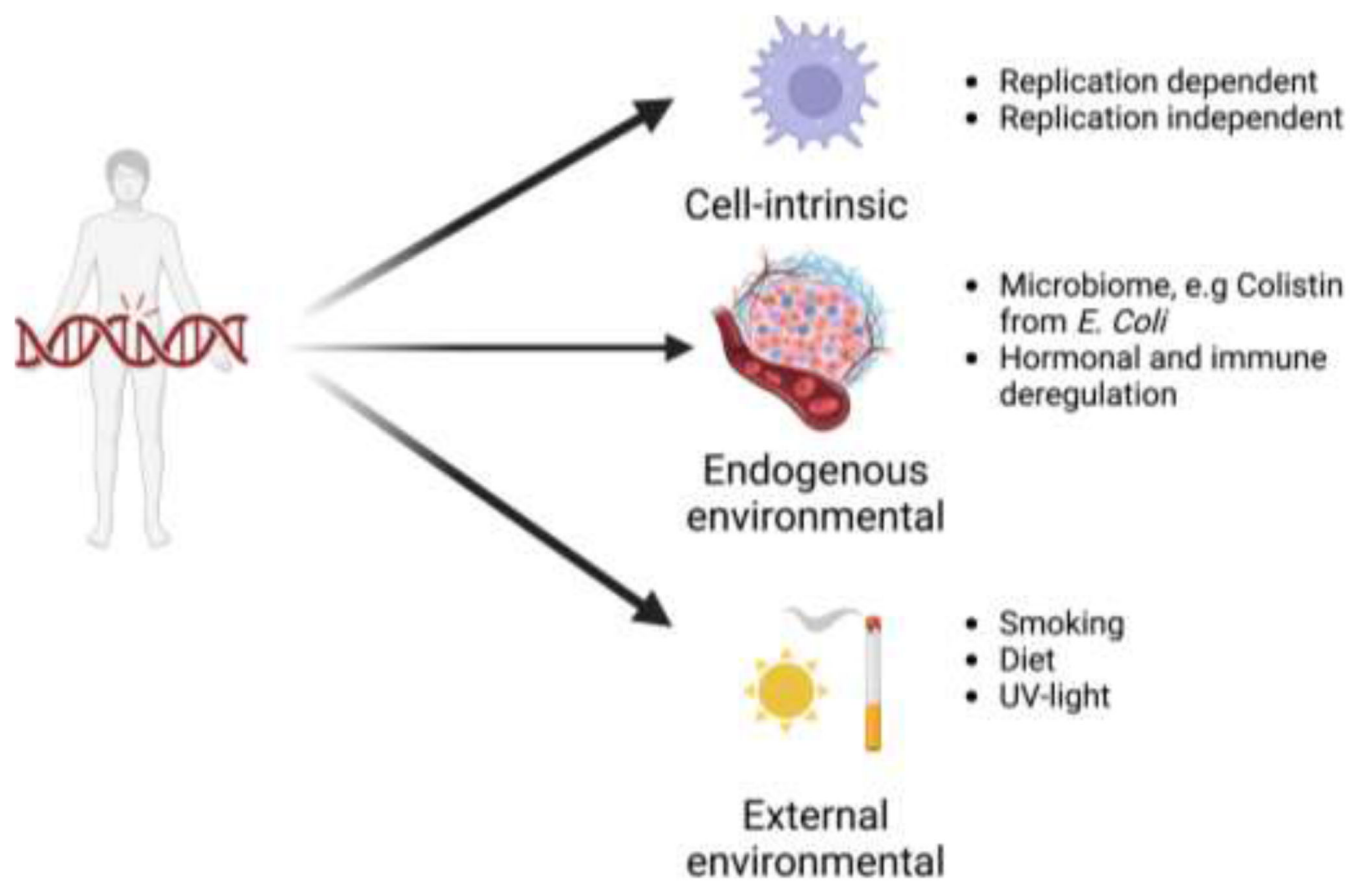
Sources of mutations in histologically normal tissues. The mutations were assessed in epithelial cells of solid organs and a variety of haematopoietic cells while assessing blood or bone marrow. The samples were derived from individuals with no known cancer diagnosis for the assessed tissue on histological examination (Created with BioRender.com).

Age-driven somatic mutations were detected in all tested human tissues, including in germ cells (24). The source of these mutations could be divided into three categories (Figure 1). The first category includes cell-intrinsic mutagenic insults. These could be cell cycle-dependent, such as those resulting from replication errors (39) or cell cycle-independent, such as those caused by spontaneous deamination of methyl cytosine and oxidative damage from endogenous sources, such as the mitochondria (33). Secondly, mutations can arise from endogenous environmental factors such as the microbiome. The microbiome is an integral part of several tissues and can contribute to somatic mutagenesis (40–42). Specific example includes colibactin produced by *Escherichia Coli* from the gut microbiome, which induces DNA-alkylation driven mutations in colon (43). Even hormones such as oestrogen can directly stimulate activation-induced deaminase function to generate somatic mutations (44). The last, and the most preventable source of somatic mutations is external environmental processes. These include smoking, ultra-violet (UV) light exposure, dietary components, and iatrogenic causes such as radiotherapy and chemotherapy (45,46). Irrespective of the source of mutagenic insult, they primarily cause single nucleotide variations (SNVs) and small insertions and deletions (INDELs) in genomes. (46–48). They can rarely also result in more complex alterations including structural chromosomal changes. Normal somatic cells accumulate roughly 9-56 SNVs/cell/year depending on the tissue-type (49). Many mutagenic processes have different probabilities to alter specific nucleotides in certain nucleotide sequence contexts, which allows deconvolution into distinct ‘mutational signatures’, each of which may represent a specific mutagenic exposure (45). The most prevalent mutational signatures found in human histologically normal somatic tissues were driven by spontaneous or enzymatic deamination of 5-methylcytosine (SBS1) and aging/oxidative damage (SBS5/40) (19). Temporal sequence can also be inferred for these mutational processes either where multiple clonally related samples are available or by timing relative to copy number gains, where early mutations increase in number according to the extent of chromosomal copy number gain upon which they are encoded, allowing identification of stepwise clonal evolutionary patterns (5,8,20–23,26,27,29,50–53). Evidence is emerging that some mutations are clonally expanded in histologically normal tissues, allowing either widespread clonal sweeps or prolonged restricted mutated clonal dominance within a single clonal unit based on local anatomy.

## Somatic mutations resulting in large clonal sweeps in histologically normal tissues

In tissues with no clear spatially restricted anatomical units, such as squamous epithelia or haematopoietic system, it is possible for a single clone with survival advantage over its neighbours to expand across a relatively large surface. This is indeed noted in the haematopoietic system, where just 12-18 clones contribute to about 30-60% of haematopoietic output in people over the age of 75 compared to more than 20,000 active clones in younger people (37). Similarly, the histologically normal urothelium in people exposed to aristolochic acid (AA) had clones measuring several square centimetres each (28). Moderately large clones measuring more than 5 square millilitres have also been identified in histologically normal oesophageal and epidermal epithelia (22,23). It is unclear what limits total clonal sweep by the dominant clone over the entire organ surface. Potentially, the timing and nature of the founder mutation, founder mutation cell-of-origin, the microenvironmental cellular and metabolic feedback loops, and the clonal competition between diverse clones could determine eventual clonal expansion (54,55). Exposure to known carcinogens also influences clonal size and diversity (23,26,28). The analysis of sun-exposed skin, urothelium in people with AA exposure history, and bronchial epithelium of smokers demonstrated that the exposure to carcinogen resulted in a higher mutational burden and a higher frequency of more than one driver mutation/clone (23,26,28). Interestingly, colonic and haematopoietic stem cells were not protected against these mutations compared to their differentiated progeny (20). The commonest of these mutations were SNVs with mutational signatures predominantly derived from spontaneous or enzymatic deamination of 5-methylcytosine (SBS1), aging (SBS5/40), and *APOBEC* activity (SBS2/13) (19). Rarely, loss of heterozygosity (LOH), copy number alterations (CNAs), and structural abnormalities were also detected, but widespread chromosomal instability (CIN) was not detected (20–37). Positively selected genes often had distinct mutations in different clones, indicating parallel evolution of clones in normal tissue. Mutations in NOTCH1 were the most frequent event in normal bronchial, oesophageal and skin epithelium (squamous epithelia), whereas mutations in epigenetic modifiers DNMT3A and KMT2D were most common in haematopoietic tissue and urothelium respectively (22,23,26,28,35,53,56–63). It is interesting to note that none of these genes is the mostly frequently mutated gene in cancers of corresponding tissues, suggesting that selection pressures in histologically normal tissues are different to those in cancer (52,53).

## Somatic mutations in histologically normal tissues where large clonal sweeps are rare

**Figure 2:**
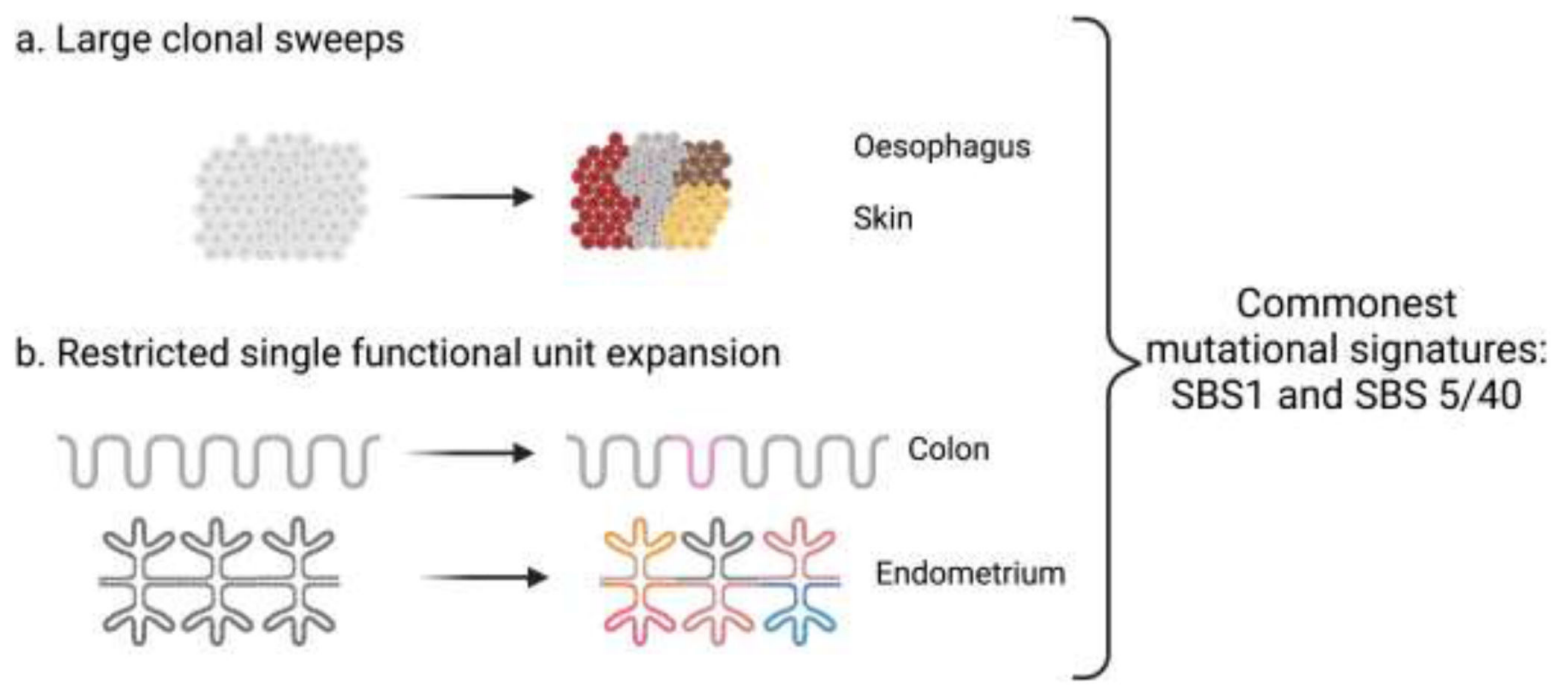
Changes in histologically normal tissues with age a. Large clones in rapidly dividing tissues without distinct anatomical units (e.g., oesophagus and skin) b. Restricted clonal size to anatomical unit in minority of tissue units (e.g., colon) or majority of tissue units (e.g., endometrium) (Created with BioRender.com).

Clonal expansions are limited in size in several tissues, likely due to histological constraints imposed by small functional units, often containing restricted stem cell pools. These include colonic crypts and endometrial glands, and post-mitotic tissues, such as neurons and smooth muscles (20,21,24,25,27,33,34,64). Positive selection of driver mutations was identified within single functional units but there were considerable variations in driver mutation frequency, for example, evidence of positive selection was noted in only 1-5% of colonic crypts and liver micro biopsies, whereas almost 90% endometrial glands in post-menopausal women were replaced by clones with positively selected driver genes (21,25,27). Interestingly, post-mitotic tissue such as neurons and smooth muscles had quantitatively similar mutations per genome per year and comparable mutational spectra with rapidly proliferating tissues such as colon, suggesting that many mutations in histologically normal tissues are replication-independent (20,33,34). Similar to tissues exhibiting large clonal sweeps most mutations were SNVs, the most frequent mutational signatures observed were SBS1 and SBS5/40, and there were differences in the relative frequency of driver gene mutations in these histologically normal tissues compared to their corresponding cancer types (20,21,24,25,33). Thus, the frequency of clones containing positively selected driver genes was variable as was their size in histologically normal tissues, but irrespectively, they shared comparable mutational signatures (Figure 2).

## Understanding the clonal and mutational overlap between histologically normal tissues and cancer

Selective pressures for alterations in driver genes in histologically normal tissues are different to those in the corresponding cancers. In the haematopoietic system, clonal expansion in haematopoietic tissue is associated with a significantly higher risk of haematological malignancies, yet no such obvious link has been noted for solid tissue malignancies (58). In fact, the likely risk of transforming a histologically normal crypt containing a positively selected driver gene into a carcinoma remains extremely low at less than 1 in 3 million over next few decades (21). There is also no direct evidence to suggest that age-matched overall mutational burden increases cancer risk. This is exemplified by the mutational burden in the small intestine, which is comparable with that of the colon at ~50 SNVs/cell/year, yet lifetime cancer risk is significantly lower in small intestine (65). This indicates the insufficiency of positively selected driver mutations and overall mutational burden in histologically normal solid tissues to significantly increase cancer risk. Precisely delineating specific bottlenecks limiting transformation of positively selected histologically normal clones and the vulnerable cell types in which these mutations occur could help differentiate between the earliest genomic aberrations required for cancer initiation from benign age-related changes which do not contribute to tumour development.

### How mutated histologically normal tissues differs from cancer

The mutational spectra and clonal dominance observed in histologically normal tissues exhibit three distinct differences compared to genomes and clonal architecture seen in cancers. Firstly, extensive CIN and whole genome duplications are rarely seen in genomes of histologically normal tissues and even LOH and single CNAs are rare (20–37). Secondly, there is a lower rate of tumour suppressor gene (TSG) inactivation in histologically normal tissues. *TP53* mutations are found in over 50% of cancers but are comparatively less frequent in histologically normal tissues, for example *TP53* mutational frequency in histologically normal oesophagus is about 25% as opposed to around 80% in oesophageal cancer (19). *PTEN* is mutated in about 80% of endometroid cancers but is mutated only in 2% of the normal endometrial glands (25,66), and *APC* mutations, an early and highly frequent event in colon cancer, are exceptionally rare in normal colonic crypts (21). Decreased selection for TSGs may be caused by a lack of CIN which in turn decreases the probability of a second hit via LOH, potentially due to lower frequency of *TP53* mutations. It is important to note that other mechanisms resulting in TSG dysfunction, such as epigenetic inactivation, within histologically normal tissues are still under explored. Thirdly, surrounding microenvironment and wild-type tissue maintain a degree of control over the extent of clonal expansion in mutated histologically normal tissues. For instance, the mouse model evidence suggests that *Trp53* mutated epidermal clones expand exponentially when exposed to UV light whereas histologically normal human epidermal clones with *TP53* mutation in UV-exposed skin remain relatively small (67). Interestingly, once UV exposure is stopped in mouse models, the survival advantage of *Trp53* mutant clones is curtailed by surrounding wild-type epidermal clones (55,67). Evidence for recolonisation of human bronchial epithelium in ex-smokers by clones carrying few tobacco-induced mutations further support the hypothesis that surrounding wild-type clones and local microenvironmental pressures can reverse the dominance of established mutant clones (26). In summary, mutated clones in histologically normal tissues have less complex genomic abnormalities, infrequently mutated tumour suppressor genes, and surrounding wild-type clones with the local microenvironment can impose a degree of control on clonal expansions.

### How mutated histologically normal tissues may facilitate cancer transformation

Clones harbouring selected driver gene mutations in histologically normal tissues could promote cancer initiation via non-cell autonomous and cell-autonomous co-operation with pre-existing/subsequent insults. Dominance by a few clones can reduce overall fitness and adaptability in histologically normal tissues, increasing its susceptibility to age-related diseases such as cancer (33,59,68–71). Moreover, some of these mutated clones may also co-operate to promote cancer initiation with neighbouring cells. At least some minor clones can facilitate proliferation of a major clone during cancer progression and treatment response in cell culture and xenotransplantation assays (72,73). Even interaction between mutated and wild-type tissue can also promote cancer initiation as suggested by mouse experiments where Notch1-mutated cells promoted cancer initiation within surrounding Notch1-wild-type cells by promoting inflammatory microenvironment akin to wound healing and by disrupting the skin barrier (74). Somatic mutations in histologically normal tissues can also potentially provide a second hit to pre-existing germline cancer susceptibility variants or could facilitate cancer initiation in conjunction with non-genetic second hits. Large scale tumour sequencing methods estimate that germline variants facilitate cancer initiation in about 2% of all cancer cases within 20 tumour types investigated (75–77). Non-genetic tumour promotor can also induce cancer initiation, as shown classically by Berenblum and Shubik in 1947 (78). They showed that the mouse skin exposed to known skin carcinogens 3:4-benzpyrene or 9:10-dimethyl-1:2-benizanthracene could only transform into frank cancer if the exposure is succeeded by croton oil application, which acted as a tumour promotor. Given that only 3/20 known or suspected common human carcinogens demonstrated discernible mutational signatures, it is possible that a wide variety of carcinogens act as a cancer promoter similar to croton oil (79). Lastly, it is conceivable that histologically normal tissues carrying mutated clones can attract an immune response, overwhelming and perhaps exhausting the immune surveillance network, and allowing cancer initiating clones to face limited scrutiny in some cases. Recent evidence suggests that pre-malignant lesions can attract such an immune response (80–82), however this possibility is unexplored in histologically normal tissues exhibiting widespread mutations and clonal expansions.

Thus, clonality in histologically normal tissue can in principle facilitate as well as inhibit cancer initiation, very likely in a context dependent manner. Assessing clonal architecture in pre-malignant lesions, understanding the cell types in which they occur and longitudinal clonal analyses from histologically normal to pre-malignant lesions could assist in identifying specific contexts in which certain clones in histologically normal tissue potentiate or abrogate cancer initiation.

## Insights from the mutational landscape of pre-malignant lesions and inferred early events during cancer initiation

**Figure 3:**
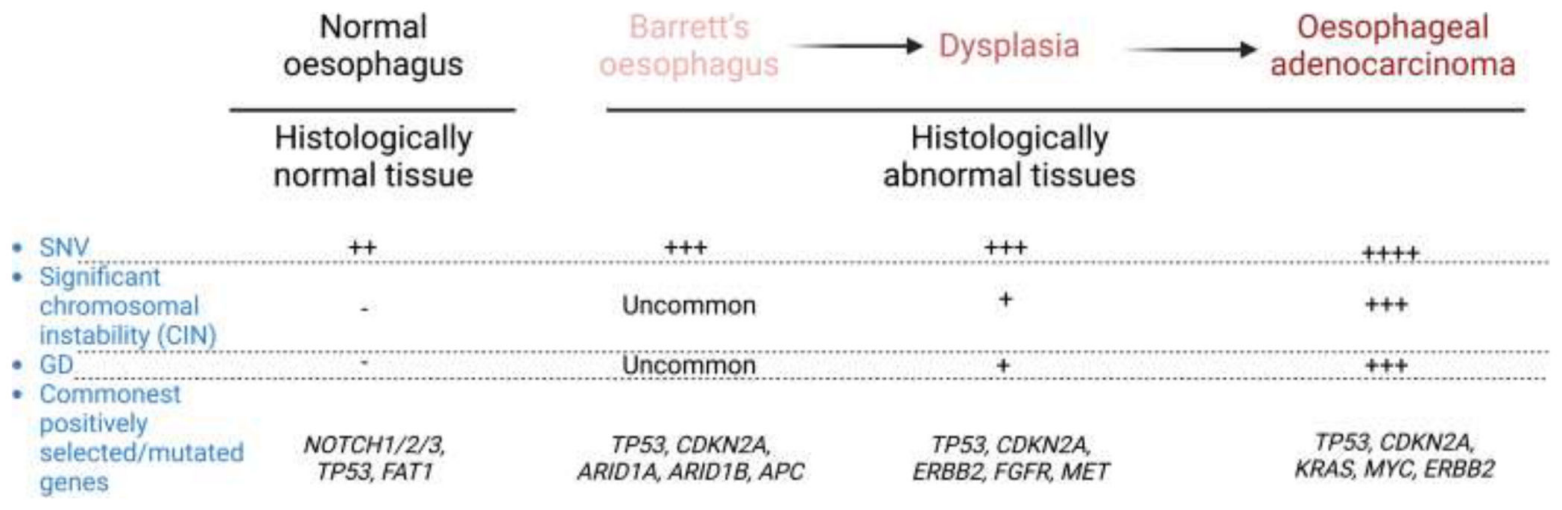
Changes in the mutational spectra in the histologically normal oesophagus and Barrett’s oesophagus compared with oesophageal dysplasia and adenocarcinoma.

Risk factors for pre-malignant lesions are heterogeneous such as acid reflux for Barret’s oesophagus, repeated scarring and regeneration for liver cirrhosis, and long-standing inflammation for inflammatory bowel diseases. Such heterogeneity in pathogenesis and variability in lead time to eventual carcinogenesis results in diverse mutational spectra and clonal evolutionary patterns. However, there are still some commonalities in their evolution. These alterations are studied in detail in the context of evolution of Barrett’s oesophagus and its further transformation into oesophageal adenocarcinoma (Figure 3) (83–86). Analysis of DNA methylation signature patterns in breast and lung *carcinoma-in-situ* suggested that epigenetic clonal mosaicism is highest just before cancer initiation (87). Specific epigenetic patterns may therefore guide fates of pre-malignant clones and their interaction with stromal/immune cells allowing cancer initiation (88,89). For example, *DNMTA3A* clonal mutation in haematopoietic cells preferentially hypomethylates polycomb repressive complex 2 targets and specific CpG motifs, favouring a myeloid fate, leading to dysregulated expression of leukaemia stem cell markers (90). It is also possible that these specific DNA methylation changes promote biallelic functional loss of tumour suppressor genes which could then drive clonal evolution in histologically normal tissue towards cancer initiation (91). In addition to epigenetic changes, some pre-malignant clones exhibit short telomeres, suggesting that activating standard or alternate telomere lengthening pathways may be necessary for cancer transformation (92,93). Some pre- malignant clones can undergo catastrophic genomic changes such as chromothripsis and kataegis in the absence of telomere maintenance (94). There is compelling evidence to suggest that these genomic changes happen very early during cancer initiation and could be the primary driver for malignant transformation (9,17,95). Complex genomic changes such as structural abnormalities, CNAs, and HLA LOH are also seen at higher frequency in pre- malignant clones (84,92,96–99). Indeed, the presence of CIN in pre-malignant lesions such as clonal haematopoiesis and Barrett’s oesophagus is associated with significantly increased risk of transformation (100–102). Further investigations suggested that cell-intrinsic factors such as HLA LOH and cell-extrinsic stromal and immune system re-education could also facilitate immune evasion permitting carcer initiation (81,97,103,104). Hence, pre-malignant lesions demonstrate specific epigenetic reprograming applying cell fate restrictions, short telomeres, more complex genetic changes, and re-educated stroma and immune system compared to mutated histologically normal tissues.

Investigating how the stroma and immune system dynamically co-evolve with age in mutated histologically normal clonal tissues and neighbouring pre-malignant lesions will be essential for a comprehensive understanding of the cancer initiation process. Preparing a ‘precancer atlas’ is one such endeavour, which aims to systematically analyse longitudinal clonal architecture of pre-malignant lesions within all major tissues at cell-intrinsic (genomic and epigenetic) and extrinsic (stromal and immune) levels (105,106).

## Implication for cancer prevention and early cancer diagnosis

**Figure 4:**
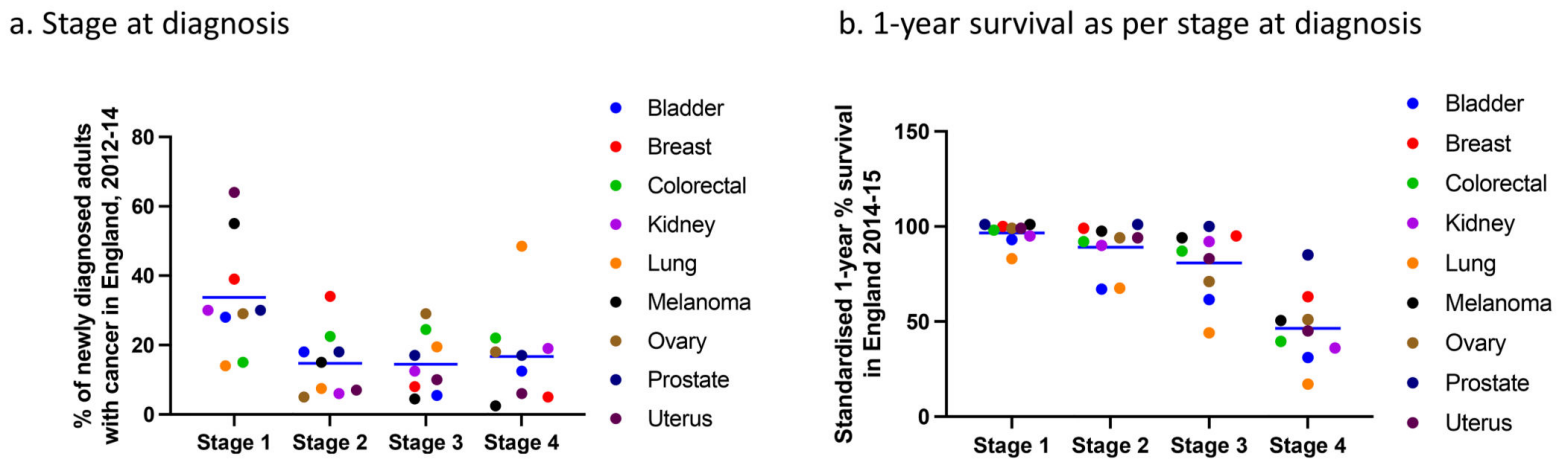
Cancer diagnosis (a) and 1-year survival as per stage (b) in common cancer diagnosed across England between 2012-14 (public health England data).

**Figure 5:**
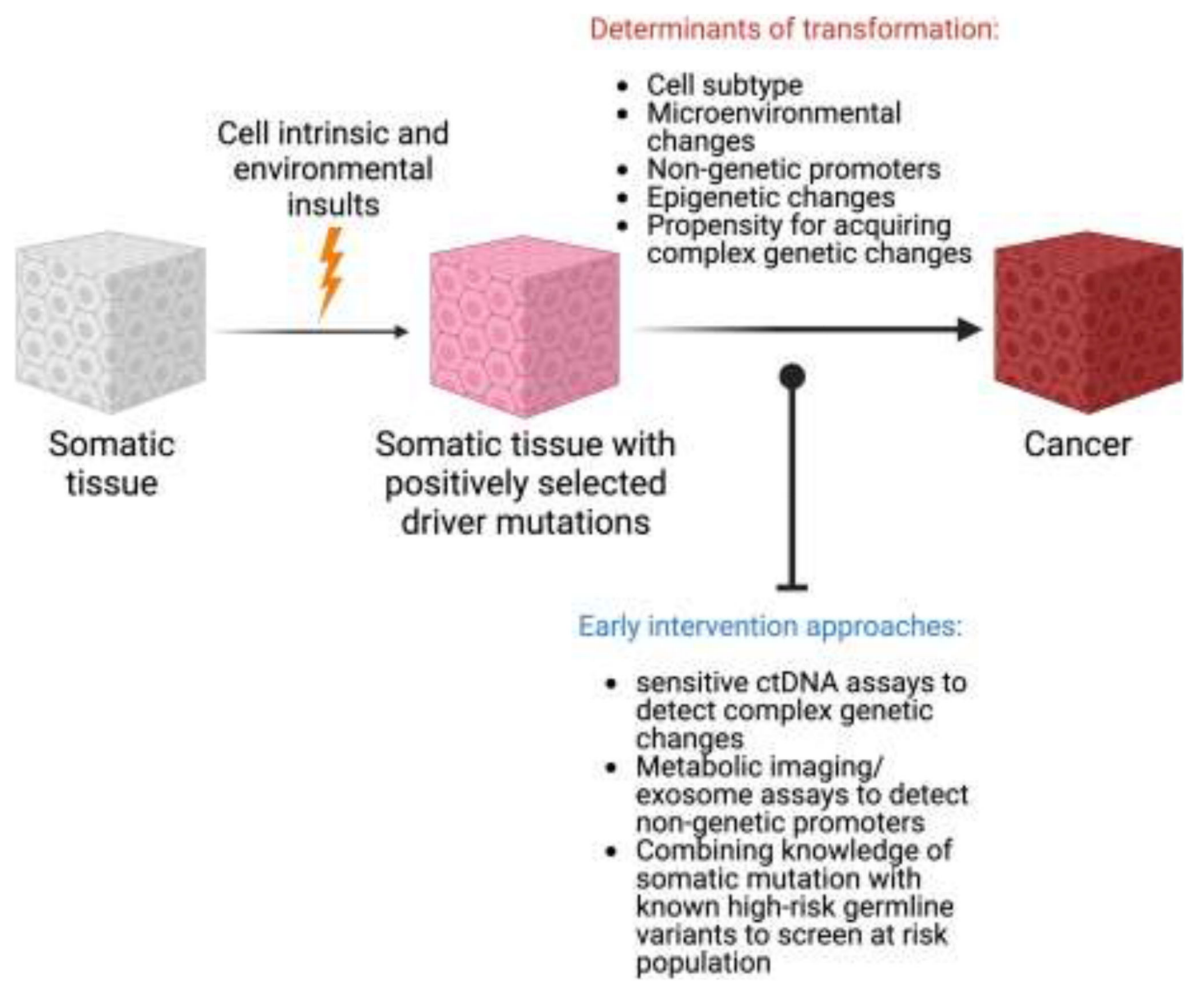
Potential determinants of carcinogenic transformation of histologically normal tissue carrying clonally expanded mutations in cancer driver genes and its application to design the early intervention strategies for cancer prevention and detection (Created with BioRender.com).

Focussing on unique changes in cancer over and above that of the mutational landscape in histologically normal tissue could be exploited for early cancer intervention. Most cancers diagnosed at an early stage (stage 1-2) have an average 1-year survival of 90-100% with some exceptions, however only about 50% of new cancers are diagnosed early in England (107) (Figure 4). One of the promising avenues to increase early cancer diagnosis by screening for circulating tumour DNA (ctDNA) (108,109). The validity of ctDNA for cancer monitoring is well-established, however using ctDNA for early cancer diagnosis remains challenging due to extremely limited ctDNA shedding at this stage, lack of tumour tissue to inform assays and requirements for ultra-high specificity (110). Exponential advances in DNA sequencing techniques and combination of mutational analysis with copy number and DNA-methylation pattern recognition in commercial tests allow detection of tumour fractions below 10^-4^ in blood with a very high specificity of 99.5% but poor sensitivity for stage 1 cancers (16.5% for DNA-sequencing approach and 39% for methylation-based analysis) in a pan-cancer setting (109–112). DNA from non-malignant clonal haemopoietic expansions can confound several ctDNA assays, however sampling of white cells in blood alongside plasma provides a convenient negative control (113). Recently, circulating cell free DNA derived from pre-malignant colonic adenomas was detected in plasma using a tumour-informed ctDNA assay suggesting future ctDNA early detection tests may be confounded by mutated DNA released from other pre-malignant or even histologically normal tissues across the body (bioRxiv 2022.01.17.476508). In such a case, targeting events specific to malignant tissues such as CNAs or specific epigenetic events may be required. There are currently limited studies focused upon DNA methylation and microenvironmental changes at the earliest stages of cancer development which may assist in refining future ctDNA assays. Our recent work has revealed that pollution activated macrophages can drive non-small cell lung cancer initiation in an *EGFR* mutant mouse model, primarily via the pro-inflammatory cytokine IL1β (Research Square 10.21203/rs.3.rs-1770054/v1). Only the AT2 cells carrying mutant EGFR could transform in the presence of IL1β in these experiments, suggesting that the fate of mutant cells in histologically normal tissue carrying positively selected mutation could be dependent on its subtype. Detection of such intermediary molecules within exosomes purified from serum/other body fluids or even via novel metabolic imaging techniques could also potentially assist in early detection and screening for cancer in specific contexts (114,115). Available data of histologically normal tissue also suggest that genomic, epigenomic, and microenvironmental changes have significant tissue-specific components which likely relate to different mechanisms of malignant progression in these tissues. Cancer-type specific early detection assays may therefore have an advantage in comparison to pan-cancer assays and high-risk populations can also usually be more readily identified for individual cancer types. Indeed, ctDNA fragmentation-based and methylation-based assays for stage A/1 liver and colon cancer detection respectively have demonstrated sensitivity of about 85% (116,117). Lastly, cancer prevention strategies could also be realised when functional determinants and promoters of clonal progression and regression during cancer initiation from histologically normal tissue would be identified. These avenues imply that somatic mutational pattern in histologically normal tissue could readily inform future cancer prevention and intervention strategies (Figure 5).

## Conclusions

The mutational spectra of histologically normal tissues are surprisingly complex with clear evidence for positive selection of cancer driver mutations. Clonal diversity decreases with age due to accumulation of driver mutations in clones that outcompete surrounding unmutated clones in proliferative tissues. Tumour initiation may begin by mutations in one such permissive clone acquiring additional genomic or epigenetic alterations with/without support from microenvironment and non-genetic promoter to acquire a pre-malignant phenotype, further increasing in clonal dominance before acquiring the next genetic or epigenetic event to become a founder cancer clone. In tissues where positively selected mutations is restricted to single functional units, increase in clonal size may not be observed until cancer initiation. Somatic mutational landscapes have now been described in more than 25 histologically normal human tissues, however outstanding questions remain about whether specific germlines SNPs promote differences in clonal architecture and clonal fate, whether similar changes are also present at epigenetic level, and whether stroma and immune system evolve differentially around each clonal expansion. Answering these questions will assist in understanding how fate of each clone is regulated and what determines cancer transformation allowing better delineation of early cancer intervention and prevention approaches.
